# Supplementary material for: Dose Escalation of Oropharyngeal Cancer: Long-Time Follow-Up and Side Effects
Source: Cancers (Basel). 2023 Apr 30;15(9):2580. doi: 10.3390/cancers15092580 (PMC10177133; doi:10.3390/cancers15092580)
Supplement: Supplementary file 1 [file cancers-15-02580-s001.zip › cancers-2231701-supplementary.pdf]

Supplementary Material

Subgroup analysis stratified by T stage

We compared OS and PFS according to prescribed dose in subgroups stratified by T-stage (Figures S1-S2). A comparison of the clinical characteristics in the subgroups, shows an imbalance in the distribution of negative prognostic factors (Table S1). In the subgroup with large primary tumours treated with standard dose radiotherapy, it is more common with older age, performance status ≥1, ongoing smoking, other primary tumour than tonsil or base of tongue cancer and HPV-negative tumours, compared to the subgroup of patients with large primary tumours treated with dose-escalated radiotherapy. All these clinical characteristics have been shown to be negative prognostic factors in oropharyngeal cancer [10, 20, 25, 26]. As the distribution of negative prognostic factors was strongly skewed towards the control group, an analysis of the influence of prescribed dose was considered impossible in our dataset.

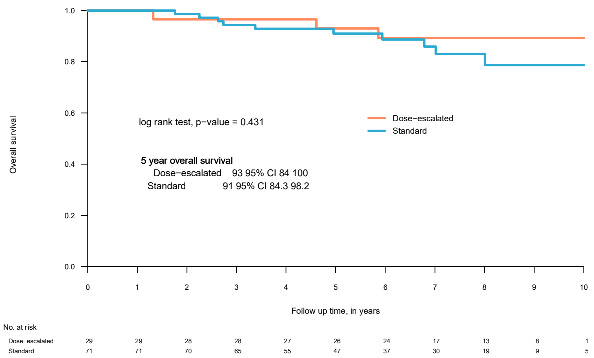

(**a**)

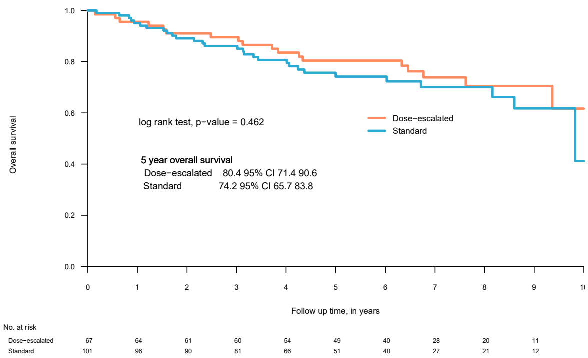

(**b**)

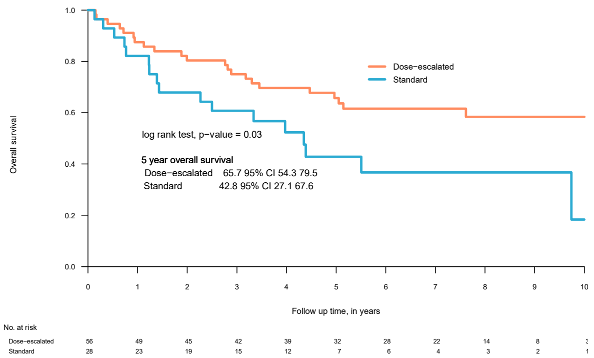

(c)

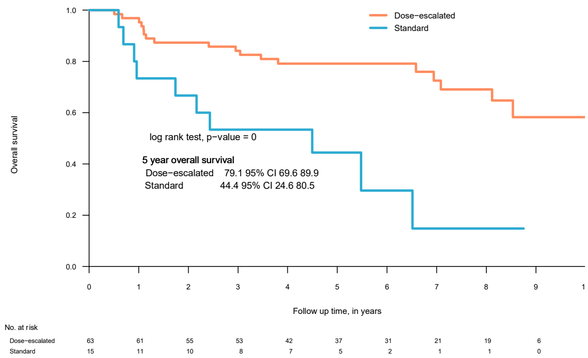

(d)

Figure S1a-d. Overall survival in subgroups stratified by T-stage. T1 (a), T2 (b), T3 (c) and T4 (d).

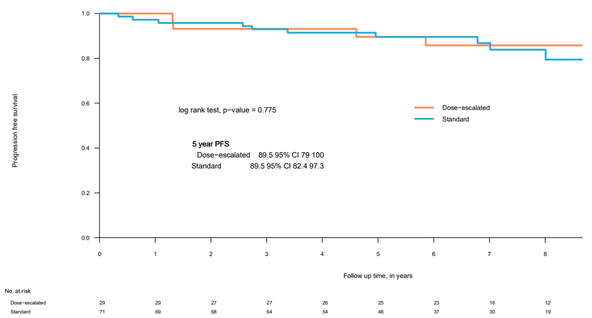

(**a**)

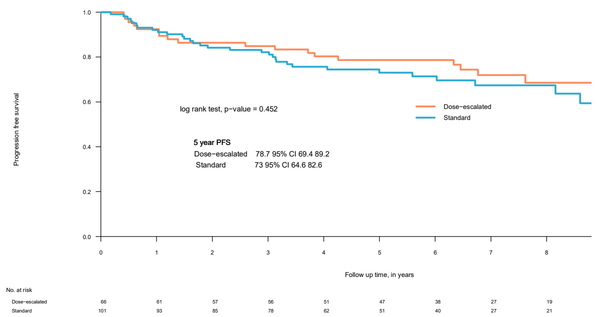

(**b**)

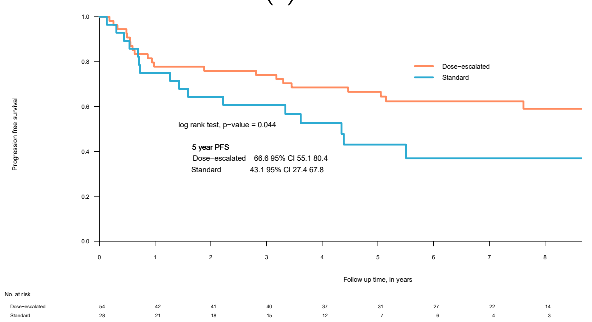

(c)

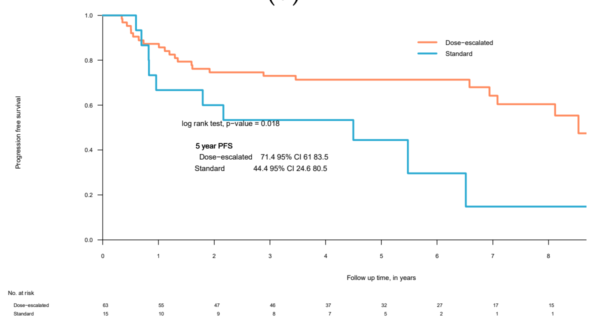

(d)

Figure S2a-d. Progression-free survival in subgroups stratified by T-stage. T1 (a), T2 (b), T3 (c) and T4 (d).

Table S1. Clinical characteristics of subgroups divided by dose level and T-stage. The skewed distribution of negative prognostic factors is highlighted in the dose-escalated cohort (green) and in the standard dose cohort (orange).

	Dose escalated				Standard dose			
	T1_T2		T3_T4		T1_T2		T3_T4	
	n	%	n	%	n	%	n	%
**Age (median)**	63		63		64		67	
**Gender (male)**	73	76	89	75	121	70	28	65
**Performance status**								
PS 0	87	91	96	81	157	91	24	56
PS ≥1	9	9	23	19	15	9	19	44
**Smoking status**								
Never	35	36	35	29	55	32	10	23
Former	42	44	53	45	89	52	13	30
Current	19	20	31	26	28	16	20	47
**Tumour type**								
Tonsillar	19	20	70	59	145	84	21	49
Base of tongue	76	79	44	37	23	13	10	23
Other	1	1	5	4	4	2	12	28
**N-status**								
N0	17	18	23	19	15	9	12	28
N1	7	7	9	8	25	15	8	19
N2a	9	9	6	5	9	5	1	2
N2b	53	55	53	45	108	63	14	33
N2c	10	10	26	22	14	8	7	16
N3	0	0	2	2	1	1	1	2
**Boost modality**								
SIB	30	31	73	61	n/a	n/a	n/a	n/a
Brachy	66	69	46	39	n/a	n/a	n/a	n/a
**HPV-status**								
Positive	81	84	101	85	157	91	18	42
Negative	15	16	18	15	15	9	25	58
**Concurrent medical treatment**								
None	27	28	20	17	36	21	13	30
Cisplatin	22	23	43	36	45	26	19	44
Cetuximab	45	47	51	43	91	53	11	26
Other	2	2	5	4	0	0	0	0
**Recurrence**								
None	85	89	85	71	154	90	30	70
Local	7	7	14	12	2	1	8	19
Locoregional	2	2	3	3	3	2	2	5
Distant metastases	1	1	9	8	11	6	2	5
Distant + locoregional	0	0	6	5	2	1	0	0
Progression during RT	0	0	0	0	0	0	1	2
Not assessable	1	1	2	2	0	0	0	0

Table S2. Clinical characteristics of patients in matched dose-escalated cohort with local recurrence

	Number	Percent
**Patients with local recurrence**	21	10
**Gender**		
Male	15	71
Female	6	29
**Tumour site**		
Tonsil	9	43
Base of tongue	11	52
Other	1	5
**T-stage categories**		
T1	0	0
T2	7	34
T3	7	34
T4	7	33
**Dose-escalation modality**		
SIB	10	48
Brachytherapy boost	11	52
**Cisplatin as part of treatment**		
Yes	10	48
No	11	52
**HPV-status**		
Positive	15	71
Negative	6	29
**Smoking status**		
Current	10	48
Former	7	33
Never	4	19
**Performance status at start of RT**		
PS 0	17	81
PS 1	3	14
PS 2	1	5
